# Influence of moisture content, temperature, and time on free fatty acid in stored crude palm oil

**DOI:** 10.1038/s41598-022-13998-1

**Published:** 2022-06-14

**Authors:** Samuel Emebu, Omokaro Osaikhuiwuomwan, Aleksi Mankonen, Chinweike Udoye, Charity Okieimen, Dagmar Janáčová

**Affiliations:** 1 Department of Automatic Control and Informatics, Tomas Bata University, Jižní Svahy Nad Stráněmi 4511, 76001 Zlin, Czech Republic; 2 Department of Chemical Engineering, University of Benin, PO Box 1154, Benin City, Nigeria; 3 Department of Energy, Lappeenranta-Lahti University of Technology, Mukkulankatu 19, 15210 Lahti, Finland; 4 Institute for Systemic Inflammation Research, University of Lubeck, Ratzeburger Allee 160, 23562 Lubeck, Germany

**Keywords:** Chemistry, Chemical engineering

## Abstract

Consequent to the importance of crude palm oil (CPO) to global food processing industries, and the need for quality assurance of CPO. A kinetic model that describes changes of free fatty acid (FFA) in industrially stored CPO has been developed. CPO FFA is a well-known indicator of the deterioration of CPO. The effect of initial moisture content, storage temperature, and time on CPO FFA have been investigated in this work. Specifically, statistical multi-regression models for changes in FFA and moisture content (MC) were developed at *P*-value < 0.05 or 95% confidence interval fence. It was found that CPO FFA increases with an increase in moisture content, temperature, and time in their linear term and in respect to decreases in their quadratic term, and interaction between moisture content and temperature. The CPO MC was also found to decrease with an increase in temperature and time and increases in the quadratic term of temperature. Although while the model for CPO FFA, based on Fisher's F-test: ${\mathrm{F}}_{\mathrm{model}}(6.80)<{\mathrm{F}}_{95\mathrm{\%}}(19.30)$, showed no lack-of-fit; that of CPO MC showed lack-of-fit, ${\mathrm{F}}_{\mathrm{model}}(13.67)\nless {\mathrm{F}}_{95\mathrm{\%}}(4.39)$. Furthermore, based on inference from the statistical model, their kinetic models were also developed. While the CPO FFA kinetic, found to be a half-order kinetic model and its other auxiliary models showed a very good fit (R^2^ {0.9933–0.8614} and RMSE {0.0020–3.6716}); that of CPO MC was a poorly fitted first-order kinetic model (R^2^ {0.9885–0.3935} and RMSE {0.0605–17.8501}).

## Introduction

Palm oil, the world's most-produced vegetable oil^1^ is commonly used in its crude or refined form in the food processing industry. It contributes significantly to the gross domestic product (GDP) of Indonesia, Malaysia, Guatemala, Nigeria, and Brazil^2^. Commercially, crude palm oil (CPO) is normally produced in large quantities by wet (i.e. water enhanced) extraction from its fruits, stripped from fresh fruit bunches (FFB)^3^. As an illustration, the Okomu oil palm company processes 60 tonnes of FFB/hr (i.e. approximately 13.2 tonnes of CPO per hour, for a 22% efficient extraction^4^ via this method^5^. This large-scale production is essential to tackle the demand for CPO. Furthermore, to also tackle the seasonal variation of FFB harvest, produced CPO are stored in fixed roof tanks^6^ with installed moisture extractor, within a moderate temperature to keep the viscosity reasonably low^7^. Normally CPO fed from the production line at approximately 90 ℃ to the storage tank, is cooled down naturally and sustained within 35–55 ℃ by a heat exchanger. The wet extraction process conditions ( i.e. ~ 90–140 ℃ and moisture content (MC) >> 3%^8^) and storage temperature facilitate an increase in the CPO free fatty acid (FFA), due to hydrolysis of various triglyceride, diglyceride, and monoglyceride molecules. These molecules are built with glycerols attached to about 50% saturated fatty acid (mostly ~ 44% palmitic and 5% stearic acid), 40% monounsaturated fatty acids (mostly oleic acid), and 10% polyunsaturated fatty acids (linoleic acids)^9^. The hydrolysis of these triglyceride molecules contributes in different portions to the increase of FFA^10^. However, low FFA value (2–5%), among other standards (such as moisture and impurity content (0.15–0.30%), bleachability index (2.1–2.8), colour (Orange–red), etc.^8,11–14^) is an important quality assurance standard for global sales of CPO. Usually FFA, moisture, and impurity content are an indicator of the other standards^15^. Therefore, to prevent the deterioration of CPO beyond the standard FFA value, stored CPO is continuously dried with the mist extractor and sold quickly.

It has been reported that FFA formation is influenced by the MC and temperature of stored vegetable oil and lipids^16^. Zhang et al.^10^ reported that as temperature increases, the percentage degradation of CPO triglyceride constituents to FFA is such that triglyceride with, linoleic > stearic > oleic > palmitic acid. Although it should be noted that there are more triglycerides with palmitic chains than other triglyceride constituents, and as such palmitic acid significantly influences CPO FFA. Almeida et al.^17^, and Taluri et al.^18^ also reported that storage temperature influenced the FFA of CPO and olive oil respectively. Furthermore, Lin et al.^19^ reported a kinetic model for FFA formation in lipid extracted from stored almonds, with consideration of the influence of relative humidity, and temperature. Although having highlighted from literature that the MC and temperature of stored vegetable oil increase FFA value, there are however no reports on a kinetic model to predict CPO FFA changes based on these two highlighted storage factors. This model when developed would facilitate the simulation, monitoring, and control of FFA in industrially stored CPO. The dynamics of FFA in vegetable oil can be investigated through the reactants (i.e. reaction of glyceride and water molecules), Eq. (1)^20–24^. However, the measurements in this approach require the simultaneous analysis of glycerides, water, and FFAs, using expensive and complex equipment such as Gas Chromatography, Liquid Chromatography, and HPLC^25–27^. Therefore a simplified approach based on the product (i.e. change in CPO FFA, $\mathrm{\%}\Delta \mathrm{FFA}$ via titration method), Eq. (2) is considered in this work. Furthermore, the model development approach for Eq. (1) is suggestive to be more complex than Eq. (2), as it can constituent multiple subequations of the hydrolysis process^20^, and as such more variables. Where, ${\mathrm{r}}_{\mathrm{FFA}}$ is the reaction kinetic, ${\mathrm{y}}_{\mathrm{FFA}}=\mathrm{\%}\Delta \mathrm{FFA}$, $\mathrm{k}$ is the reaction constant, $\mathrm{n}$ is the reaction order, $\mathrm{i}$ is the specific glyceride molecule considered, ${\mathrm{x}}_{\mathrm{gly},\mathrm{i}}$ and ${\mathrm{x}}_{\mathrm{water}}$ are the compositions of glycerides, and water in the CPO.

1 $${\mathrm{r}}_{\mathrm{FFA},\mathrm{i}}={\mathrm{kx}}_{\mathrm{gly},\mathrm{i}}{\mathrm{x}}_{\mathrm{water}}$$

2 $${\mathrm{r}}_{\mathrm{FFA}}=\mathrm{k}{\Delta {\mathrm{y}}_{\mathrm{FFA}}}^{\mathrm{n}}$$

Therefore, in this work, CPO FFA and MC with known initial FFA and MC stored at different temperatures in a surrounding of known relative humidity will be analysed at different storage times. The objectives shall involve: Analysis and collection of adequate quantity of CPO of specific MC from commercial CPO processing company; Design of experimental via Box–Behnken design (BBD) of the minimum and maximum limit of factors (i.e. initial MC (with known measured FFA), temperature and time) and with final FFA and MC as responses; Storage of the CPO samples at the designed BBD condition, and subsequent evaluation of the specified responses; Deduce statistical multi-regression model for the designed BBD, and evaluation of the factors and equations at 95% confidence level using the Fisher's F-test; Evaluation of FFA and MC kinetics and deduction of an n^th^ order kinetic model.

## Materials and methods

### Collection of the source sample

CPO samples were selected from the regular sampling routine of a CPO processing company based on the acceptable maximum limit (0.20%) and range (0.015–0.30%) of MC in edible vegetable oil^14^. Three sample sets of 0.20 ± 0.02%, 0.25 ± 0.02%, and 0.30 ± 0.02% MC were identified, and their FFA were also determined (5.17, 5.05, and 4.28% respectively). Thereafter adequate quantity (1000 ml) of these samples were collected, sealed, and stored as a source sample in an enclosure with an Ultraviolet-C radiation lamp to limit FFA increase as reported by Said et al.^28^. Table 1 shows the properties of the CPO source samples given by the company.

**Table 1:** Average physicochemical properties of crude palm oil source sample.

Properties, unit	Result
Density, 40 °C/20 °C H_2_O	0.90
Viscosity, cP	41.3
Saponification value, mgKOH/g oil	201.2
Unsaponification value, g/kg	3.8
Peroxide value, mEq O_2_/kg oil	4.0
Iodine value, Wijs	48.1
Dirt content, wt%	0.024
colour	Red (25–30)–Orange (26–28)

### Determination of free fatty acid

Based on Eq. (3)^29,30^, 2 g of CPO sample, ${\mathrm{m}}_{\mathrm{CPO}}$, was mixed with 10 mL of ethyl alcohol. The mixture was heated to about 35 °C, cooled, and titrated with 0.1 N NaOH solution, ${\mathrm{N}}_{\mathrm{NaOH}}$, via 2–3 drops of phenolphthalein until a colour change to pale pink, and the volume of the titrant noted, ${\mathrm{V}}_{\mathrm{T}}$. Where 25.6 is the equivalence factor for palmitic acid—the dominant fatty acid in CPO^31^. The hydrolysis of palm oil, based on glyceryl palmitate,${\mathrm{C}}_{19}{\mathrm{H}}_{38}{\mathrm{O}}_{4}$ to palmitic acid, ${\mathrm{C}}_{16}{\mathrm{H}}_{32}{\mathrm{O}}_{2}$ is given by Eq. (4).

3 $$\mathrm{\%FFA}=25.6{\mathrm{V}}_{\mathrm{T}}{\mathrm{N}}_{\mathrm{NaOH}}/1.99{\mathrm{m}}_{\mathrm{CPO}}$$

4 $${\mathrm{C}}_{19}{\mathrm{H}}_{38}{\mathrm{O}}_{4}+{\mathrm{H}}_{2}\mathrm{O}\to {\mathrm{C}}_{3}{\mathrm{H}}_{8}{\mathrm{O}}_{3}+{\mathrm{C}}_{16}{\mathrm{H}}_{32}{\mathrm{O}}_{2}$$

After the initial measurement of FFA for the source sample, ${\mathrm{FFA}}_{\mathrm{i}}$, subsequent FFA of samples at different temperatures and storage times, ${\mathrm{FFA}}_{\mathrm{t}}$, are expressed in terms of percentage increase to the ${\mathrm{FFA}}_{\mathrm{i}}$ of the sample, Eq. (5).

5 $$\mathrm{\%}\Delta \mathrm{FFA}=100{\mathrm{FFA}}_{\mathrm{t}}/{\mathrm{FFA}}_{\mathrm{i}}$$

### Determination of moisture content

Following the American Oil Chemists’ Society (AOCS) air oven (Ca 2c-25) method^32^ based on Eq. (6), 10 g of CPO sample, ${\mathrm{m}}_{\mathrm{CPO},\mathrm{i}}$, measured via a sensitive weighing balance was dried at a given temperature and time in an oven operating at ~ 70% relative humidity (RH). It was allowed to cool in desiccators for 15 min and the final weight of the sample, ${\mathrm{m}}_{\mathrm{CPO},\mathrm{f}}$ noted.

6 $$\mathrm{\%MC}= 100({\mathrm{m}}_{\mathrm{CPO},\mathrm{i}}-{\mathrm{m}}_{\mathrm{CPO},\mathrm{f}})/{\mathrm{m}}_{\mathrm{CPO},\mathrm{i}}$$

Note that the initial or total $\mathrm{MC}$ of the source sample, ${\mathrm{MC}}_{\mathrm{i}}$ was deduced by heating at 105 ℃ for 4 h. Subsequent $\mathrm{MC}$ for samples at different temperatures and storage times, ${\mathrm{MC}}_{\mathrm{t}}$, are expressed in terms of percentage reduction of ${\mathrm{MC}}_{\mathrm{i}}$ of a specific sample, Eq. (7).

7 $$\mathrm{\%}\Delta \mathrm{MC}=100{\mathrm{MC}}_{\mathrm{t}}/{\mathrm{MC}}_{\mathrm{i}}$$

### Statistical model analysis

Design of experiment via BBD was implemented to determine whether MC, temperature, and storage time indeed influence the CPO FFA as reported in literature. This was achieved by the deduction of multi-variable regression model using Python Jupyter Notebook, and was also deduced for CPO MC.

#### Design of experiment

A “three-variable-three-level” BBD of three (3) centre points was adopted resulting in fifteen (15) experimental runs, $\mathrm{N}$, with the three specified variables considered for each response. The design analyses the contribution of these variables in terms of linear, quadratic, and interaction effects in the prediction of CPO FFA and MC via a generalised second-order polynomial regression model, Eq. (9). Equation (8) and Table 2 shows the relationship between coded, $\mathrm{x}$ and actual values, $\upxi$ of the specified variables for the analysis. Where $\overline{\upxi }=$ 0.5 $({\upxi }_{\mathrm{max}}+ {\upxi }_{\mathrm{min}})$, ${\upxi }_{\mathrm{max}}$ and ${\upxi }_{\mathrm{min}}$ are the mean, maximum and minimum actual value of the experiment.

**Table 2:** Coded and actual levels of the process variables for the Box–Behnken design.

Variable, unit	Symbols	Coded and actual value		
		− 1, lower limit	0, midpoint	1, upper limit
Moisture content, %	${x}_{1}$	0.20	0.25	0.30
Temperature, °C	${x}_{2}$	35	60	85
Time, h	${x}_{3}$	6	27	48

8 $$\mathrm{x}=\left(\upxi -\overline{\upxi }\right)/0.5({\upxi }_{\mathrm{max}}-{\upxi }_{\mathrm{min}})$$

In a similar manner for which the three sample sets for CPO have been chosen (i.e., based on the CPO MC standard for edible vegetable oil). The upper and lower limit of temperature has also been chosen based on the suggested range at which CPO is processed and stored by commercial CPO processing companies.

9 $${\text{Y}} = {\upbeta }_{0} + \Sigma _{{{\text{i}} = 1}}^{\mathbbm{n}} {\upbeta }_{{\text{i}}} {\text{x}}_{{\text{i}}} + \Sigma _{{{\text{i}} = 1}}^{{\mathbbm{n}}} \upbeta _{{{\text{ii}}}} x_{{\text{i}}}^{2} + \Sigma _{{{\text{i}} = 1}}^{{\mathbbm{n}}} \Sigma _{{{\text{j}} = 1}}^{{\mathbbm{n}}} \upbeta _{{{\text{ij}}}} {\text{x}}_{{\text{i}}} {\text{x}}_{{\text{j}}}$$

Where $\mathrm{Y}$, is the response, i.e. $\mathrm{\%}\Delta \mathrm{FFA}$ or $\mathrm{\%}\Delta \mathrm{MC}$ as given by Eqs. (5) and (7) respectively. ${\mathrm{x}}_{\mathrm{i}}$ and ${\mathrm{x}}_{\mathrm{j}}$ represent the specified variables of consideration, ${\upbeta }_{0}$ is the constant of the model, ${\upbeta }_{\mathrm{i}}$ is the linear term coefficient,${\upbeta }_{\mathrm{ii}}$ is the quadratic term coefficient, ${\upbeta }_{\mathrm{ij}}$ is the interaction coefficient and $\mathbbm{n}$ is the number of variables considered.

### Reaction kinetics analysis

The reaction kinetics for the CPO FFA and MC was deduced using Eq. (10), by curve fitting experimental data of FFA and MC for the three source samples collected. 50 g of each of these source samples was poured into a 50 ml beaker of height, 60.96 mm and diameter, 38.10 mm. The sample was left opened and placed in an oven, and the analysis of the sample’s FFA and MC following the procedures described earlier were performed at a temperature interval of 10 ℃ from 35 to 85 ℃, and at a time interval of 6 h from 6 to 120 h. The rate constant, ${\rm{k}}$ for the reaction kinetics were deduced on the assumption that the reaction order (i.e.,$\mathrm{n}$, the power factor on $\mathrm{\%}\Delta \mathrm{FFA}$, ${\mathrm{y}}_{\mathrm{FFA}}$ and $\mathrm{\%}\Delta \mathrm{MC}$, ${\mathrm{y}}_{\mathrm{MC}}$) is within zero-to-second order (i.e., $\mathrm{n}$ = 0, 0.5, 1.0, and 2.0).

10 $${\mathrm{r}}_{\mathrm{j}}={\mathrm{dy}}_{\mathrm{j}}/\mathrm{dt}=\pm {\mathrm{ky}}_{\mathrm{j}}^{\mathrm{n}}$$

In modelling FFA kinetics, ${\mathrm{r}}_{\mathrm{FFA}}$, it is assumed $\mathrm{k}$ is a function of temperature, and change in initial moisture content, $\Delta {\mathrm{MC}}_{\mathrm{i}}$, such that it is a product of the Arrhenius relationship based on temperature and $\Delta {\mathrm{MC}}_{\mathrm{i}}$ (Where $\Delta {\mathrm{MC}}_{\mathrm{i}}={\mathrm{MC}}_{\mathrm{i}}-\Delta {\mathrm{MC}}_{\mathrm{i},0.20\mathrm{\%}}$ i.e. changes in MC of a sourced sample from the standard 0.20% MC) as expressed by Eqs. (11) and (12).

11 $$\mathrm{k}={\mathrm{k}}_{0}{\mathrm{e}}^{-\mathrm{E}/\mathrm{RT}}$$

12 $${\mathrm{k}}_{\mathrm{m}}={\mathrm{k}}_{\mathrm{m}0}{\mathrm{e}}^{\mathrm{\varphi }\Delta {\mathrm{MC}}_{\mathrm{i}}/273\mathrm{R}}$$

Equation (11) is deduced by curve fitting the value of $\mathrm{k}$ computed for each CPO source sample at different temperatures based on the results from Eq. (10). While ${\mathrm{k}}_{\mathrm{m}}$, Eq. (12) is deduced by curve fitting the value of the ratio of ${\mathrm{k}}_{0}$ in Eq. (11) with respect to ${\mathrm{k}}_{0}$ of the maximum allowable 0.20% MC, i.e. ${\mathrm{k}}_{\mathrm{m}}={\mathrm{k}}_{0}/{\mathrm{k}}_{\mathrm{0,0.20\%}}$. This manipulation implies reexpressing $\mathrm{k}$ as Eq. (13). Where R is the ideal gas constant, 8.314 J·mol^−1^·K^−1^, $\mathrm{T}(\mathrm{K})$ is the temperature, $\mathrm{E}$(J·mol^−1^) is the activation energy, $\mathrm{\varphi }$(J·mol^−1^) is activation energy due to $\Delta {\mathrm{MC}}_{\mathrm{i}}$, and 273 is the temperature in Kelvin at standard temperature and pressure.

13 $$\mathrm{k}={\mathrm{k}}_{\mathrm{m}}{\mathrm{k}}_{\mathrm{0,0.20\%}}{\mathrm{e}}^{-\mathrm{E}/\mathrm{RT}}$$

In modelling MC kinetic, ${\mathrm{r}}_{\mathrm{MC}}$, Eqs. (10) and (11) are the only applicable equations to utilise.

#### Evaluation of deduced kinetic model

The goodness-of-fit for the kinetic models deduced were evaluated using R-squared (R^2^), Eq. (14) and Root mean squared error (RMSE), Eq. (15). The use of these two evaluation criteria is hinged on the report that R^2^ is inappropriate when used for demonstrating the performance or validity of certain nonlinear models^33^. Consequent to this report Jim^34^ has suggested that RMSE would be appropriate for such cases. On the basis that the kinetic models would be evaluated for $n$ = 0, 0.5, 1, and 2, hence the possibility of nonlinearity. Hence, for nonlinear models, RMSE would be used as the main criterion even though R^2^ would also be reported. The R^2^ value ranges between 0 and 1, the closer to unity the better the model, while RMSE ranges between 0 and $\infty$, the closer to zero the better. Where $\mathrm{y}$ and $\overline{\mathrm{y} }$ is the output and mean output of the experimental data,$\widehat{\mathrm{y}}$ is the curve fitted model output, $\mathrm{N}$ is the number of the experimental run, and $\mathrm{p}$ is the number of dependent variables in the model.

14 $${\mathrm{R}}^{2}=1-\frac{{\Sigma \left(\mathrm{y}-\widehat{\mathrm{y}}\right)}^{2}}{{\Sigma \left(\mathrm{y}-\overline{\mathrm{y} }\right)}^{2}}$$

15 $$\mathrm{RMSE}=\sqrt{\frac{{\Sigma \left(\mathrm{y}-\widehat{\mathrm{y}}\right)}^{2}}{\mathrm{N}-\mathrm{p}-1}}$$

The curve fitting of kinetic models was implemented using Lsqcurvefit curve-fitting tools in MATLAB.

## Results and discussion

### Result of design of experiment

Before developing stored CPO FFA and MC kinetic models, statistical analysis via the Box–Behnken design of experiment was used to investigate the variables that significantly influence them. This analysis was achieved by comparison of coefficients, ${\upbeta }_{0}, {\upbeta }_{\mathrm{i}}$, ${\upbeta }_{\mathrm{ii}}$ and ${\upbeta }_{\mathrm{ij}}$ computed with *P*-value ** < 0.05 or 95% confidence interval fence. The resulting multi-regression models for the statistical analysis are given by Eqs. (16) and (17). The model shows, FFA increases with an increase in initial moisture content, ${\mathrm{x}}_{1}$, temperature, ${\mathrm{x}}_{2}$, and time, ${\mathrm{x}}_{3}$ in their linear term and decreases in their quadratic term, and interaction between moisture content and temperature, ${\mathrm{x}}_{1}{\mathrm{x}}_{2}$. This result is quite similar to Gawrysiak-Witulska et al.^35^ reported on the effect of moisture content and temperature on the degradation of phytosterol, which is also found in lipid. In the Gawrysiak-Witulska et al.^35^ report a three factorial analysis of variance of the factors given in Table 2 was also performed, but only in respect to linear (${\mathrm{x}}_{1}$, ${\mathrm{x}}_{2}$, and ${\mathrm{x}}_{3}$), and interactive terms (${\mathrm{x}}_{1}{\mathrm{x}}_{2}$, ${\mathrm{x}}_{1}{\mathrm{x}}_{3}$, ${\mathrm{x}}_{2}{\mathrm{x}}_{3}$ and ${\mathrm{x}}_{1}{\mathrm{x}}_{2}{\mathrm{x}}_{3}$), and it was found that moisture content, temperature, storage time significantly influenced the degradation of rapeseed phytosterol in all highlighted terms.

Furthermore, CPO MC in reference to Eq. (17) decreased with an increase in the linear terms of temperature, ${\mathrm{x}}_{2}$, and time,${\mathrm{x}}_{3}$, and increased with an increase in the quadratic term of temperature, ${\mathrm{x}}_{2}^{2}$.

16 $${\mathrm{y}}_{\mathrm{FFA}}=112.50+1.50{\mathrm{x}}_{1}+3.50{\mathrm{x}}_{2}+1.5{\mathrm{x}}_{1}{\mathrm{x}}_{2}-3.25{\mathrm{x}}_{1}^{2}-2.75{\mathrm{x}}_{2}^{2}-2.25{\mathrm{x}}_{3}^{2}$$

17 $${\mathrm{y}}_{\mathrm{MC}}=24.30-41.94{\mathrm{x}}_{2}-7.25{\mathrm{x}}_{3}+23.26{\mathrm{x}}_{2}^{2}$$

**Figure 1:**
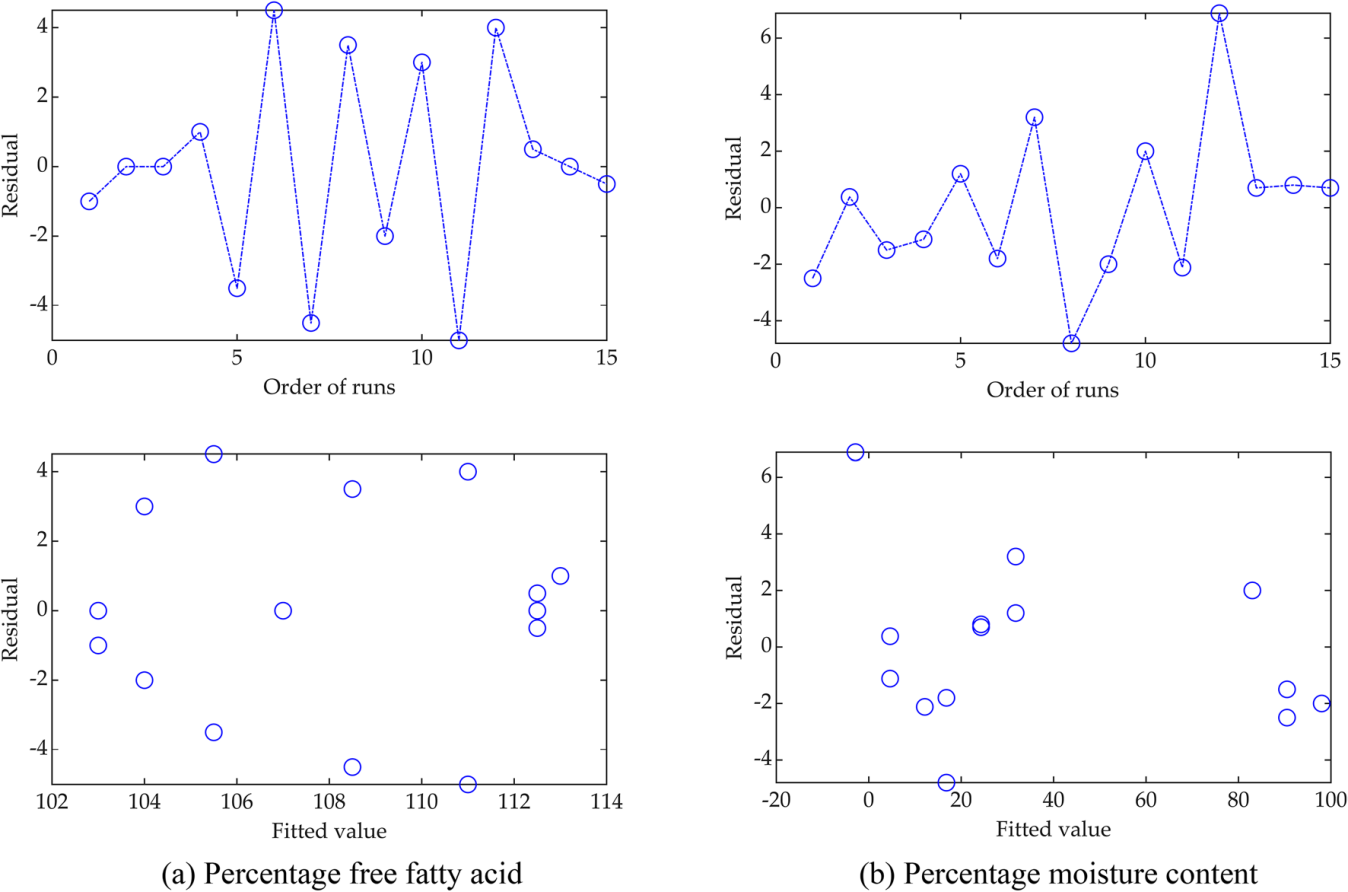
Residual plot of the statistical multi-regression model.

Although these equations showed the significant variables and their relationship to FFA and MC. While ${\mathrm{y}}_{\mathrm{FFA}}$ showed no lack-of-fit, i.e. ${\mathrm{f}}_{\mathrm{model}}(6.80)<{\mathrm{f}}_{95\mathrm{\%}}(19.30)$, ${\mathrm{y}}_{\mathrm{MC}}$ showed lack-of-fit, i.e. ${\mathrm{f}}_{\mathrm{model}}(13.67)\nless {\mathrm{f}}_{95\mathrm{\%}}(4.39)$, as such ${\mathrm{y}}_{\mathrm{FFA}}$ is adequate for prediction of FFA, as opposed to ${\mathrm{y}}_{\mathrm{MC}}$ for prediction of MC. ${\mathrm{y}}_{\mathrm{FFA}}$ having passed the Fisher's F-test, further evaluation of its adequacy is performed by the residual plots, Fig. 1. Observation of the plot showed no adequate signs of block effect and the variance does not increase in trend. This is an indication that the Box–Behnken design is well randomised. Therefore, it can be inferred that ${\mathrm{y}}_{\mathrm{FFA}}$ can be used to predict FFA for various values of the variables within their specified minimum and maximum limits.

**Figure 2:**
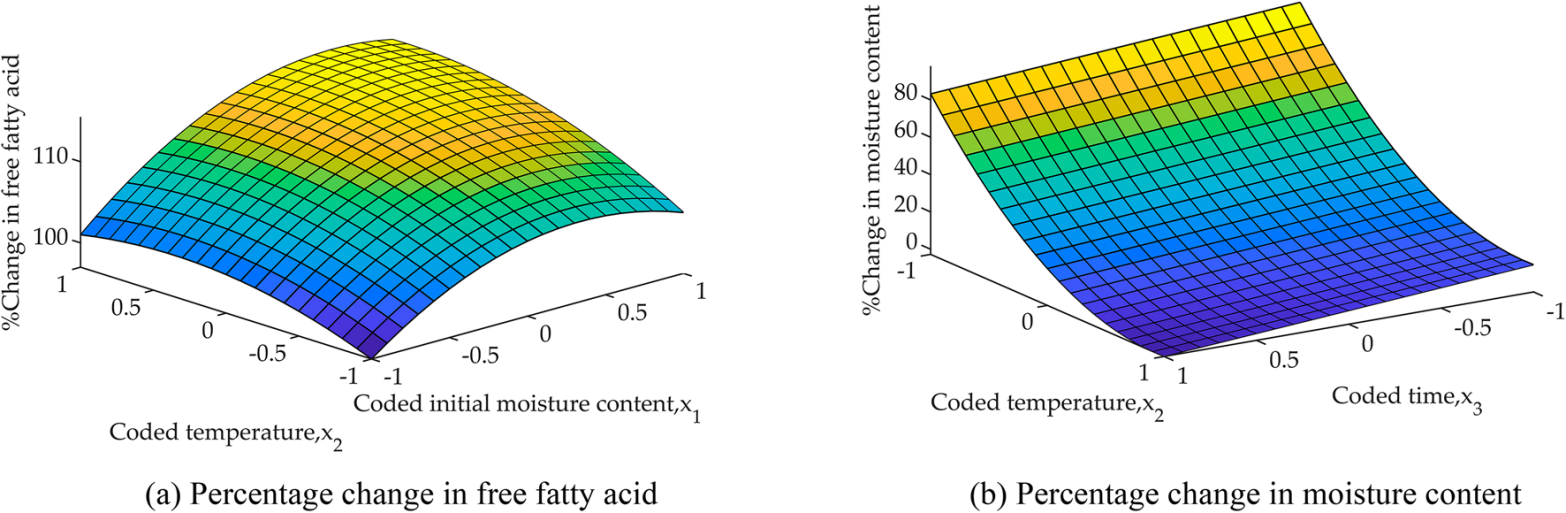
Response surface plot of the statistical multi-regression model.

The resulting response surface plot for ${\mathrm{y}}_{\mathrm{FFA}}$ within specified limits of the process variable is shown in Fig. 2. Although ${\mathrm{y}}_{\mathrm{MC}}$ has not passed the F-test, its plot is also shown. Analysis of Eq. (16) and Fig. 2a, shows, CPO FFA increases with ${\mathrm{x}}_{1}$, ${\mathrm{x}}_{2}$, and ${\mathrm{x}}_{3}$, and Eq. (17) and Fig. 2b indicate MC in CPO decreases with ${\mathrm{x}}_{2}$, and ${\mathrm{x}}_{3}$.

Based on the discussion thus far, it can be inferred that in developing the CPO FFA kinetics, the effect of moisture content, and temperature should be incorporated into the model. And as would be expected temperature would be used for CPO MC kinetics.

### Result for reaction kinetic

#### Reaction kinetic for CPO FFA

**Figure 3:**
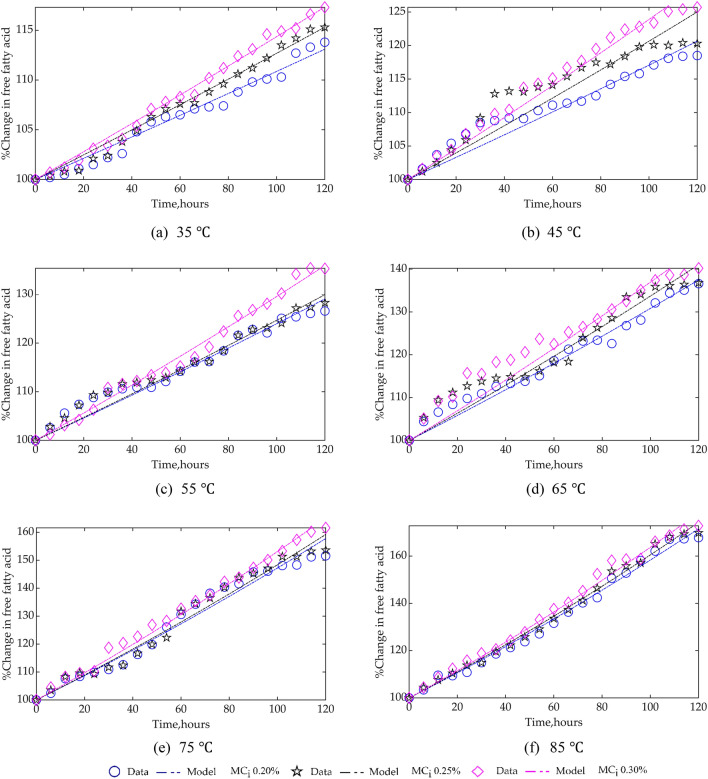
Comparison of experimental data and kinetic model of CPO FFA at different temperatures and ${\mathrm{MC}}_{\mathrm{i}}.$

The result of curve-fitting experimental data to the kinetic model, Eq. (10) via trial-and-error of reaction order, $n$ = 0.0, 0.5, 1.0 and 2.0 showed half-order reaction kinetics, $n$ = 0.5 fits the model best. Comparison of experimental data and deduced model is shown in Fig. 3, and Table 3, which highlights the estimated rate constant, $\mathrm{k}$, evaluation criteria ${\mathrm{R}}^{2}$ and RMSE for the model. Based on the ${\mathrm{R}}^{2}$ value, the best curve fit was for 0.30% ${\mathrm{MC}}_{\mathrm{i}}$ and 35 ℃ and 0.25% ${\mathrm{MC}}_{\mathrm{i}}$ and 85 ℃. While for RMSE, 0.25%, 0.30% ${\mathrm{MC}}_{\mathrm{i}}$ and 35 ℃ was the best.

**Table 3:** Rate constant for CPO FFA kinetics ($\mathrm{n}$ = 0.5, i.e., half-order reaction).

Temperature, °C	Moisture content, 0.20%			Moisture content, 0.25%			Moisture content, 0.30%		
	$\mathrm{k},{\mathrm{h}}^{-1}$	${\mathrm{R}}^{2}$	RMSE	$\mathrm{k},{\mathrm{h}}^{-1}$	${\mathrm{R}}^{2}$	RMSE	$\mathrm{k},{\mathrm{h}}^{-1}$	${\mathrm{R}}^{2}$	RMSE
35	0.0106	0.9762	0.7293	0.0123	0.9883	0.6077	0.0139	0.9933	0.4904
45	0.0164	0.9247	1.6765	0.0197	0.8614	2.7166	0.0226	0.9746	1.4449
55	0.0227	0.9592	1.8065	0.0233	0.9655	1.7277	0.0277	0.9883	1.2673
65	0.0288	0.9761	1.8230	0.0313	0.9369	3.1107	0.0341	0.9249	3.6716
75	0.0427	0.9705	3.1514	0.0436	0.9765	2.9010	0.0475	0.9915	1.9529
85	0.0516	0.9916	2.1385	0.0533	0.9933	1.9393	0.0559	0.9904	2.4421

**Figure 4:**
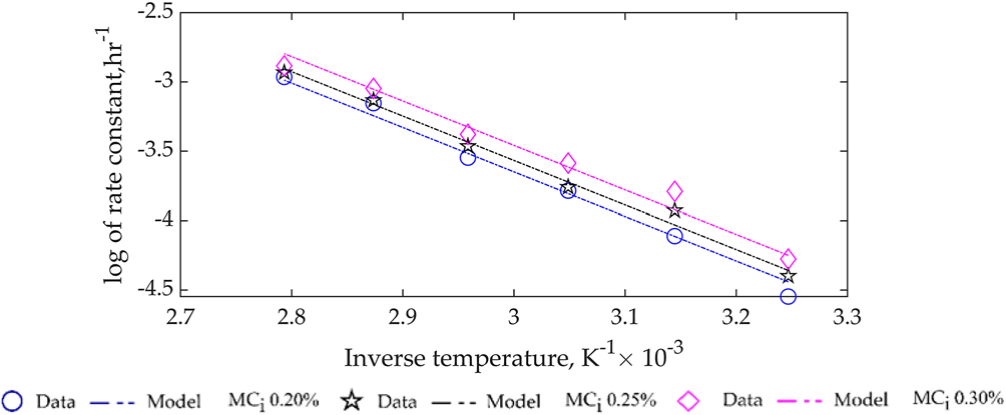
Variation of rate constant for CPO FFA kinetics with temperature for different ${\mathrm{MC}}_{\mathrm{i}}.$

In general, from the tread in the values of $\mathrm{k}$, as storage time progresses, CPO FFA increases with higher ${\mathrm{MC}}_{\mathrm{i}}$ and temperature. This observation is also clearly indicated in Fig. 4.

Figure 4, was deduced from the curve-fit of the rate constants, $\mathrm{k}$, in Table 3 to Eq. (11), and the resulting Arrhenius constants,${\mathrm{k}}_{0}$, activation energy, $\mathrm{E}$, and evaluation criteria, ${\mathrm{R}}^{2}$ and RMSE are given in Table 4. Analysis of the ${\mathrm{R}}^{2}$ and RMSE values, indicate the model has a very good fit with the rate constants, $\mathrm{k}$.

**Table 4:** Arrhenius constants and activation energy based on rate constant dependency on temperature.

Moisture content	${\mathrm{k}}_{0}{\mathrm{h}}^{-1}$	$\mathrm{E},\mathrm{J}.{\mathrm{mol}}^{-1}$	${\mathrm{R}}^{2}$	RMSE
0.20%	388.95	26,641.03	0.9877	0.0733
0.25%	422.37		0.9889	0.0634
0.30%	470.83		0.9771	0.0860

**Figure 5:**
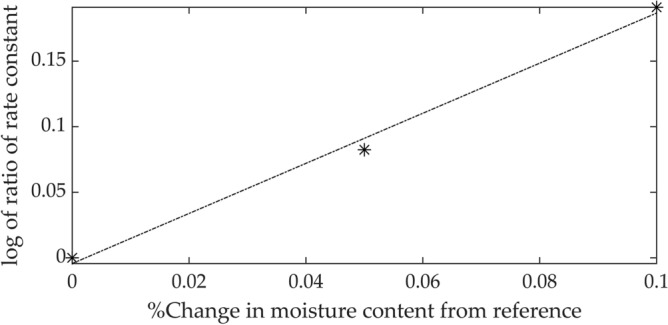
Variation of rate constant ratio, ${k}_{m}$, with changes in initial moisture content, $\Delta {\mathrm{MC}}_{\mathrm{i}}$.

Furthermore, to account for the effect of moisture content, the deduced Arrhenius constants, ${\mathrm{k}}_{0}$, in Table 4 was curve fitted to Eq. (12) via the ratio, ${\mathrm{k}}_{\mathrm{m}}={\mathrm{k}}_{0}/{\mathrm{k}}_{\mathrm{0,0.20\%}}$ and $\Delta {\mathrm{MC}}_{\mathrm{i}}$. The resulting Arrhenius constants, ${\mathrm{k}}_{\mathrm{m}0}$, activation energy to $\Delta {\mathrm{MC}}_{\mathrm{i}}$, $\mathrm{\varphi }$, and evaluation criteria, ${\mathrm{R}}^{2}$ and RMSE are given in Table 5. Observation of Fig. 5, and as supported by the ${\mathrm{R}}^{2}$ and RMSE values, the model shows a very good fit with ${\mathrm{k}}_{\mathrm{m}}$.

**Table 5:** Arrhenius constants and activation energy based on rate constant dependency on moisture content.

Moisture content	${\mathrm{k}}_{\mathrm{m}}={\mathrm{k}}_{0}/{\mathrm{k}}_{\mathrm{0,0.20\%}}$	$\Delta {\mathrm{MC}}_{\mathrm{i}}$	${\mathrm{k}}_{\mathrm{m}0}$	$\mathrm{\varphi },\mathrm{J}.{\mathrm{mol}}^{-1}$	${\mathrm{R}}^{2}$	RMSE
0.20%	1.0000	0.0000	0.9956	4335.93	0.9956	0.0107
0.25%	1.0859	0.0500				
0.30%	1.2105	0.1000

The final curve-fitted model for CPO FFA kinetics is given by Eq. (18) as deduced from Eq. (10) and (13), noting that the point of initialisation for the model is 100%.

18 $${\mathrm{r}}_{\mathrm{FFA}}={\mathrm{ky}}_{\mathrm{FFA}}^{0.5}={(0.9956\mathrm{e}}^{4335.93\Delta {\mathrm{MC}}_{\mathrm{i}}/273\mathrm{R}}{{)(\mathrm{k}}_{\mathrm{0,0.20\%}}\mathrm{e}}^{-26641.03/\mathrm{RT}}){\mathrm{y}}_{\mathrm{FFA}}^{0.5}$$

The modelling approach and results for CPO FFA kinetics, Eq. (18) described thus far is quite similar to the results reported by Lin et al.^19^ for FFA kinetics of extracted oil from stored almonds. However, in Lin et al.^19^ report, first-order reaction kinetics were proposed with consideration of the influence of relative humidity (RH), and temperature. The reported data indicated that FFA formation was more temperature-dependent and the reaction rate increased faster at higher RH than at lower RH. The RH in Lin et al.^19^ report can be assumed to have the same effect as the initial moisture content, ${\mathrm{MC}}_{\mathrm{i}}$ used in this work, since RH was kept constant in this case. The inference on the effect of temperature and RH in Lin et al.^19^ report can be observed from the increasing rate constant, $\mathrm{k}$ with temperature in Table 3 and ${\mathrm{k}}_{0}$ with ${\mathrm{MC}}_{\mathrm{i}}$ in Table 4 respectively. Furthermore, it should be noted that as opposed to the consideration of modelling rate constant as a function of initial moisture content, i.e. ${\mathrm{k}}_{0}=\mathrm{f}({\mathrm{MC}}_{\mathrm{i}})$ and keeping the activation energy, $\mathrm{E}$ constant in the Arrhenius model, Eq. (11). Lin et al.^19^ estimated both variables as a function of RH.

#### Reaction kinetic for CPO MC

**Figure 6:**
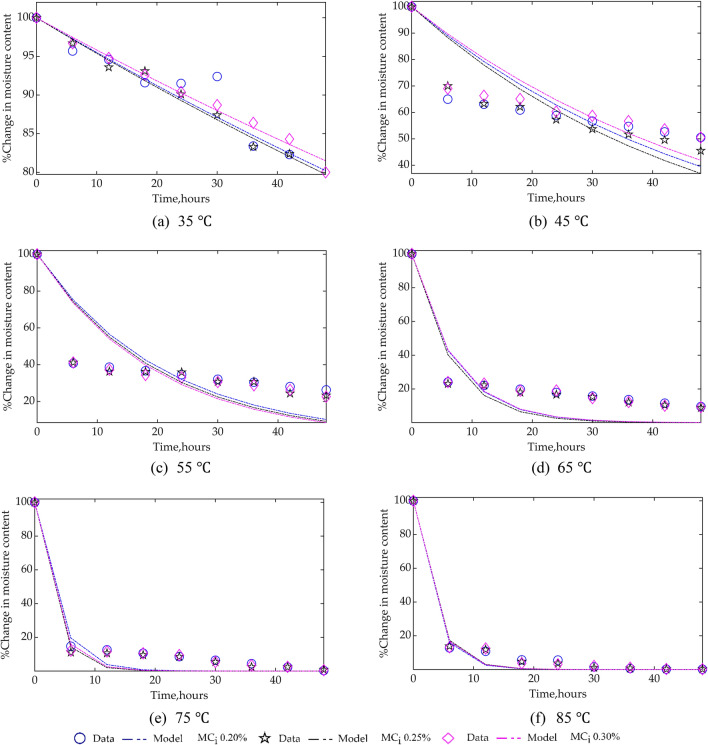
Comparison of experimental data and kinetic model of CPO MC at different temperatures and ${\mathrm{MC}}_{\mathrm{i}}.$

Similar to the CPO FFA kinetics, CPO MC kinetics were also investigated. The resulting curve-fit of experimental data to the proposed kinetic model, Eq. (10), showed a first-order reaction kinetic, $n$ = 1.0 fits the model best. The comparison of experimental data and kinetic model are shown in Fig. 6, and Table 6, which highlights the estimated rate constant,$\mathrm{k}$, evaluation criteria ${\mathrm{R}}^{2}$ and RMSE for the model. Based on the ${\mathrm{R}}^{2}$ value (linearity holds), the model gave better fits at lower (i.e., 35 ℃) and higher (75 and 85 ℃) temperatures across the three ${\mathrm{MC}}_{\mathrm{i}}$ samples. Furthermore, the value of $\mathrm{k}$ is observed to be highest for 0.25% ${\mathrm{MC}}_{\mathrm{i}}$, and lowest for 0.20% ${\mathrm{MC}}_{\mathrm{i}}$, contrary to the expectation that $\mathrm{k}$ would have consistently changed proportionally with changes in ${\mathrm{MC}}_{\mathrm{i}}$ or remained constant. This inconsistency is indicative of the fact that the proposed kinetic model for the CPO MC is inadequate, as observed from the visual inspection of Fig. 6. The reason for this poor result can be attributed to the fact that during the drying of moisture in liquid samples, the moisture is constantly in equilibrium with the moisture in the surrounding air. Therefore, if the rate of moisture removal is not high enough (i.e., at low temperature) small amount of moisture is removed, while at a higher temperature a lot of moisture is removed. And in between low and high temperatures, the rate of moisture removal is quite inconsistent. In addition, factors such as amount and effective or exposed surface area of samples, and variation of the relative humidity of the surrounding can significantly affect the rate of moisture removal.

**Table 6:** Rate constant for CPO MC kinetics ($\mathrm{n}$ = 1.0, i.e. first-order reaction).

Temperature, °C	Moisture content, 0.20%			Moisture content, 0.25%			Moisture content, 0.30%		
	$\mathrm{k},{\mathrm{h}}^{-1}$	${\mathrm{R}}^{2}$	RMSE	$\mathrm{k},{\mathrm{h}}^{-1}$	${\mathrm{R}}^{2}$	RMSE	$\mathrm{k},{\mathrm{h}}^{-1}$	${\mathrm{R}}^{2}$	RMSE
35	0.0046	0.8951	2.5419	0.0047	0.9670	0.8621	0.0043	0.9867	0.7809
45	0.0194	0.3935	12.8891	0.0208	0.6690	10.4147	0.0181	0.5502	10.9637
55	0.0475	0.4588	17.8501	0.0497	0.5162	17.3243	0.0513	0.5363	17.0494
65	0.1416	0.8552	13.7170	0.1518	0.8783	12.9938	0.1400	0.8617	13.2828
75	0.2718	0.9752	6.8943	0.3256	0.9809	6.3305	0.3062	0.9762	6.9163
85	0.3063	0.9877	4.4630	0.2924	0.9885	4.1643	0.2974	0.9875	4.3401

**Figure 7:**
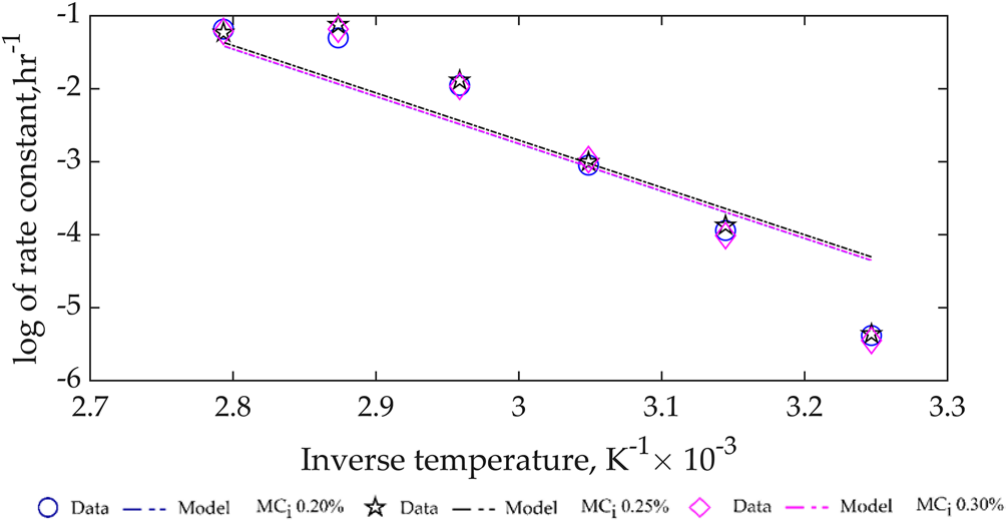
Variation of rate constant for CPO MC kinetics with temperature for different ${\mathrm{MC}}_{\mathrm{i}}.$

Furthermore, the rate constants, $\mathrm{k}$, in Table 6 was curve-fitted as a function of temperature using the linearised form of Eq. (11), and the results are given in Table 7 and based on the ${\mathrm{R}}^{2}$ value the model showed poor fit, as also illustrated in Fig. 7. In general, the poor fit of the CPO MC kinetic model, Eq. (10) and (11), collaborate with the result of lack-of-fit of the statistical multi-regression model, Eq. (17) earlier developed.

**Table 7:** Arrhenius constants and activation energy based on rate constant dependency on temperature.

Moisture content	${\mathrm{k}}_{0}, {\mathrm{h}}^{-1}$	$\mathrm{E},\mathrm{J}.{\mathrm{mol}}^{-1}$	${\mathrm{R}}^{2}$	RMSE
0.20%	${1.7844\times 10}^{7}$	53,903	0.8639	0.6807
0.25%	${1.8731\times 10}^{7}$		0.8508	0.7196
0.30%	${1.7892\times 10}^{7}$		0.8478	0.7391

The curve-fitted CPO MC kinetics from the combination of Eqs. (10) and (11) is given by Eq. (19) with an initialisation of 100%.

19 $${\mathrm{r}}_{\mathrm{MC}}=-{\mathrm{ky}}_{\mathrm{MC}}=-{{\mathrm{k}}_{0}\mathrm{e}}^{-53903/\mathrm{RT}}{\mathrm{y}}_{\mathrm{MC}}$$

By comparison of the CPO MC kinetics with the Thin-layer drying curve equations, Eq. (19), when algebraically integrated, is equivalent to the Henderson and Pabis model^36^. Having earlier highlighted the inadequacy of CPO MC kinetics, there is a need to deduce an elaborate model to predict the changes of CPO MC with temperature, following comprehensive approaches described in literature^37–39^.

## Conclusion

In conclusion, the result of this work showed mathematically how moisture content, ${\mathrm{x}}_{1}$, temperature, ${\mathrm{x}}_{2}$, and storage time, ${\mathrm{x}}_{3}$ influenced changes in CPO FFA from the analysis of a statistically significant multi-regression model, deduced from the Box–Behnken design of experiment for these process factors. It was found that each of these factors significantly influenced CPO FFA linearly (${\mathrm{x}}_{1}$, ${\mathrm{x}}_{2}$ and ${\mathrm{x}}_{3}$), quadratically (${\mathrm{x}}_{1}^{2},$ ${\mathrm{x}}_{2}^{2}$ and ${\mathrm{x}}_{3}^{2}$) and by an interaction between moisture content and temperature (${\mathrm{x}}_{1}{\mathrm{x}}_{2}$) only. Additionally, a well-fitted half-order reaction kinetic model was developed to describe the changes of CPO FFA with the incorporation of the effect of temperature and moisture content via the Arrhenius model. Similarly, a multi-regression and first-order kinetic model was developed for CPO MC. However, the multi-regression was statistically insignificant, and the first-order kinetic model had a poor fit with experimental data. The inadequacy of CPO MC models developed may suggest that elaborate considerations of physical and thermodynamic phenomena associated with the evapouration of moisture from CPO needs to be further investigated.

## Supplementary Information

Supplementary Information.

## Data Availability

The authors do not wish to share supplementary data, because all vital experimental data are the same as the data used for the plots in this article.
